# Microbial, chemical, and isotopic monitoring integrated approach to assess potential leachate contamination of groundwater in a karstic aquifer (Apulia, Italy)

**DOI:** 10.1007/s10661-024-12477-6

**Published:** 2024-02-27

**Authors:** L. Beneduce, F. Piergiacomo, P. P. Limoni, L. E. Zuffianò, M. Polemio

**Affiliations:** 1 Department of the Science of Agriculture, Food, Natural Resources and Engineering (DAFNE), University of Foggia, Via Napoli, 25 -71122 Foggia, Italy; 2 Present address: Faculty of Science and Technology, Free University of Bolzano-Bozen, Piazza Università 1, 39100 Bolzano-Bozen, Italy; 3 CNR-IRPI, National Research Council, Research Institute for Hydrogeological Protection, Via Amendola 122/I, 70126 Bari, Italy

**Keywords:** Landfill, Leachate, Groundwater, Microbial monitoring, Isotope

## Abstract

Landfill sites are subjected to long-term risks of accidental spill of leachate through the soil and consequential contamination of the groundwater. Wide areas surrounding the landfill can seriously be threatened with possible consequences to human health and the environment. Given the potential impact of different coexisting anthropic pollution sources (i.e., agriculture and cattle farming) on the same site, the perturbation of the groundwater quality may be due to multiple factors. Therefore, it is a challenging issue to correctly establish the pollution source of an aquifer where the landfill is not isolated from other anthropic land uses, especially in the case of a karstic coastal aquifer. The present study is aimed at setting in place an integrated environmental monitoring system that included microbiological, chemical, and isotope methods to evaluate potential groundwater pollution in a landfill district in the south of Italy located in Murgia karstic aquifer. Conventional (microbial plate count and physical–chemical analyses) and advanced methods (PCR-ARISA, isotope analysis of δ^18^O, δ^2^H, ^3^H, δ ^13^C, δ ^15^N-NO_3_^−^, and δ ^18^O-NO_3_^−^) were included in the study. Through data integration, it was possible to reconstruct a scenario in which agriculture and other human activities along with seawater intrusion in the karst aquifer were the main drivers of groundwater pollution at the monitored site. The microbiological, chemical, and isotope results confirmed the absence of leachate effects on groundwater quality, showing the decisive role of fertilizers as potential nitrate sources. The next goal will be to extend long-term integrated monitoring to other landfill districts, with different geological and hydrogeological characteristics and including different sources of pollution, to support the ecological restoration of landfills.

## Introduction

Among potential groundwater pollution sources, landfills still represent a major threat. The highly toxic landfill leachate could accidentally or chronically leak from the disposal site and reach the underlying soil and the saturated aquifer, representing a serious pollution source (Abd El-Salam and I. Abu-Zuid 2015). Despite UN Sustainable Development Goals clearly stating a call for environmentally sound disposal facilities, the management of the existing landfill sites still poses many challenges. Even though modern landfills for urban solid waste are designed to minimize environmental impacts (Feng et al., 2020), when they are improperly built (i.e., without engineered liners and/or leachate collection/purification systems) or poorly managed, the risks of underground leachate infiltration increase consequently (Feng et al., 2020; Kjeldsen et al., 2002; Negi et al., 2020).

Evidence of groundwater contamination by landfill leachate has been found in many regions of the world (Guo et al., 2022; Ringle Raja et al., 2023), and researchers have been working hard to develop methodology to monitoring soil pollution and groundwater contamination (Ameloko & Ayolabi, 2018; Maryadi et al., 2020).

In Italy, a new model has been proposed that can be a helpful management tool for monitoring the potential contamination process of groundwater due to the presence of landfills with municipal solid waste, including a significant organic component (Sappa et al., 2023).

Considering the general characteristics of municipal landfill wastes, landfill leachate may be defined as a water-based solution with a high content of dissolved organic matter, inorganic macro-components, heavy metals, and xenobiotic organic compounds (Christensen et al., 1994).

Groundwater is generally oligotrophic open environments that host different micro- and macro-organisms, with ecological functions in the recycling and distribution of energy and organic matter (Danielopol et al., 2003). Specifically, they host microbial communities that are particularly responsive to environmental changes, and the evaluation of their disturbances may act as bio-indicators of ecosystem health (Griebler & Avramov, 2015). Moreover, groundwater quality depends both on natural factors, such as the geochemical and hydrogeological characteristics of the aquifer, biological activity, and aquifer biodiversity, and on anthropogenic factors, mainly related to the type and concentration of released pollutants (Fida et al., 2023; Girvan et al., 2005; Loreau, 2000; McCann, 2000).

The landfill ecosystems are characterized by specific microbial communities involved in the process of waste degradation that may differ in abundance and distribution due to the type and age of landfill, the oxygen content, and switching from aerobic to facultative-anaerobic/anaerobic (from cellulose and hemicellulose hydrolyzing bacteria to methanogenic bacteria and Actinobacteria) (Kjeldsen et al., 2002; Liu et al., 2019). Also, pathogenic bacteria of human or animal origin can be found, associated with urban and farming waste disposal (Javahershenas et al., 2022).

As a direct consequence of the landfill leachate release into groundwater, the aquifer microbiota may show clear signs of disturbance, drastically shifting most of the key taxa (Gu et al., 2022; Lu et al., 2012). Complementary to microbiological monitoring, geochemical approaches that consider each potential nitrogen source and methods using isotopes (mainly oxygen, hydrogen, carbon, and nitrogen) as tracers can be used in the case of groundwater. Hydrogeochemical, isotopic, and microbiological investigations are necessary to elucidate the primary mechanism controlling the biogeochemistry of NO_3_^−^ in the groundwater environment (Wang et al., 2024).

Different isotopic compositions may indeed characterize nitrates (NO_3_^−^) and other pollutants of different origins. Specifically, the δ^15^N values range from − 4‰ to + 4‰ for synthetic fertilizers, from + 2‰ to + 5‰ for soil organic nitrogen, and from + 10‰ to + 20‰ for human and animal waste nitrate (Kendall et al., 2007). Synthetic fertilizers are also characterized by enriched ^18^O values (+ 17‰– + 25‰) (Kendall et al., 2007), while high values of tritium (^3^H) and δ^13^C are indicators of leachate impacts (Cossu et al., 2018; Wimmer et al., 2013).

This paper proposed an integrated environmental monitoring system to evaluate possible leachate contamination events of groundwater from municipal solid waste landfills using hydrogeochemistry, stable isotopes, and microbiological methods. The use of classical hydrogeological methodologies was integrated with the use of advanced molecular microbial ecology methods and chemical and isotopic analyses.

**Fig. 1:**
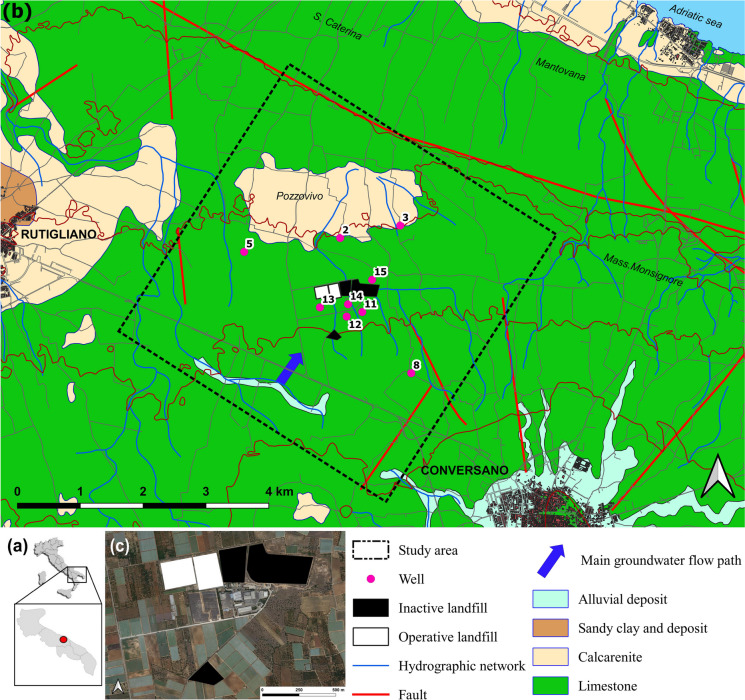
Study area maps. **a** Italy, Apulia region, and study area location. **b** Geological and hydrogeological schematic map. **c** Aerial view of the Conversano landfill district (CLD); the five landfill sites are distinguished in inactive (black) and operative (white)

We investigate the presence of pollution from leachate in groundwater in a landfill district located near the town of Conversano, in the province of Bari, the main town of the Apulia region (south-eastern Italy, Fig. 1).

The geological and hydrogeological characteristics of the area make the Murgia karst aquifer vulnerable due to the lack of adequate surface protection and expose it to a very high risk of pollution (Polemio & Limoni, 2006; Polemio et al., 2009).

The inclusion of other potential causes of karstic groundwater quality disturbances is another advantage of our integrated methodologies: public authorities and environmental monitoring agencies could easily fall into misinterpretations of data arising from conventional methods that do not allow a comprehensive scenario. This may induce an underestimation or overestimation of the potential contamination of the site.

## Hydrogeological features

The large carbonate hydrogeological structure called Murgia can be distinguished in the Apulia region (Fig. 1). It constitutes a wide coastal karstic aquifer, the high-quality groundwater of which is used for drinking. The Murgia plateau is mainly made of limestone (Mesozoic calcareous and/or calcareous-dolomitic rocks); the hydraulic conductivity is from medium to high but is heterogeneous and anisotropic for karstic and fractured features. It shows surface and deep karstic features, such as poljes, swallow holes, dolines, and dry valleys. The limestone outcrops are widespread below a very thin layer of residual soil in the study area; it is somewhere overlapped by subsequent formations, constituted by calcarenite, sandy clay, and alluvial deposits. The groundwater-saturated flow involves predominantly the limestone formation, which is part of a deep karstic aquifer, involving the whole Murgia hydrogeological structure.

These karstic features of the Murgia aquifer determine a wide range of groundwater vulnerability, from low to very high, as assessed with multiple methods applied in a test area located not far from the Conversano landfill district (CLD) (Polemio et al., 2009).

The recharge area includes inland portions of the site, and the outflow goes along the Adriatic coast and the Ionian coast. Serious seawater intrusion effects are known for this coastal aquifer (Polemio, 2016). The salinity threshold of pure fresh groundwater in the aquifer was assessed to be equal to 0.5 g/L. The threshold was defined by discussing chemical analyses of 500 groundwater samples, recognizing samples free from seawater intrusion mixing, and discussing statistically the salinity variability of this subset of samples; for these samples (defined as “pure fresh groundwater”), the salinity variability is mainly due to water-carbonate rock interaction (Polemio, 2016; Polemio et al., 2009). The coastal strip of Murgia, where the groundwater salinity is higher than the law potability limit (1.5 g/L), is about 3 km wide, as close to CLD, up to 6 km. The main human activity in the area is agriculture, and the most common crops are vineyards, orchards, olive groves, and arable crop cultivations.

The CLD includes five municipal solid waste (MSW) landfills, two of which are still operating (Zuffianò et al., 2018). The local groundwater flow is toward the Adriatic Sea (6 km away, north-northeast). The water table depth is so high, generally not less than 120 m from the ground surface, that it complicates any type of hydrogeological survey and sampling. Groundwater overexploitation for irrigation promotes groundwater–seawater intrusion mixing (Casarano et al., 2019; Polemio, 2016).

## Materials and methods

### Sampling

The groundwater and landfill leachate samples were taken concurrently with monthly samplings carried out in December 2017 and January 2018 in the CLD, as shown in Fig. 1.

The chemical study focused on the major ions together with some minor ions: potassium (K^+^), sodium (Na^+^), calcium (Ca^2+^), magnesium (Mg^2+^), boron (B^−^), fluoride (F^−^), bromide (Br^−^), chloride (Cl^−^), sulfate (SO_4_^2−^), ammonium (NH_4_^+^), nitrate (NO_3_^−^), nitrite (NO_2_^−^), and bicarbonate (HCO_3_^−^) to define the geochemical characteristics.

The isotope characterization of groundwater and leachate was focused on δ^18^O, δ^2^H, ^3^H, δ ^13^C, δ ^15^N-NO_3_^−^_,_ and δ ^18^O-NO_3_^−^.

During the groundwater sampling, specific measurements in the field were performed using a multiparametric probe (Hydrolab-Quanta G): electrical conductivity (EC), temperature (T), pH, dissolved oxygen (DO), and redox potential (Eh). The determination of NH_4_^+^ and NO_2_^−^ was carried out by means of a photometric field method. All water samples were collected and stored in high-density polyethylene bottles (500 mL) with watertight caps. Samples for cation analysis were acidified by the addition of nitric acid (HNO_3_^−^) to a pH < 2, while water samples for metals determination were filtered through a cellulose acetate membrane (pore size 0.45 µm) and then were acidified by HNO_3_^−^ to a pH < 2.

The sample for the dissolved carbonate δ^13^C_DIC_ was acidified with orthophosphoric acid (H_3_PO_4_), according to Atekwana and Krishnamurthy (1998). The sample for δ^15^N-NO_3_^−^ and δ^18^O-NO_3_^−^ was acidified with hydrochloric acid (HCl) to a pH < 2.

Two liters of groundwater, one used for the bacteriological counts and one for the extraction of microbial DNA, respectively, were taken from groundwater wells after 5 min of outflow and sterilization of the outlet taps by an alcohol-soaked wipe and then by a portable Bunsen burner. Groundwater samples and samples of the leachate from the landfill, collected directly from the landfill collection tank, were placed in pre-sterilized Pyrex glass bottles. Both the water and leachate samples were then transported in refrigerated bags and analyzed within 4 h for microbial counts or stored at − 20 °C for subsequent DNA extraction.

### Bacterial count

The total mesophilic and *Escherichia coli* bacterial counts were conducted according to the Italian APAT-IRSA standard methods (APAT, IRSA-CNR 2003) that refer to the APHA methods (APHA, AWWA, WEF 2005).

One hundred milliliters of each water sample was filtered under sterile conditions on 45 mm cellulose acetate membranes with a 0.45 μm pore size (Millipore). The membranes were subsequently placed on PCA (Oxoid, Basingstoke, UK) culture medium for total mesophilic bacterial counts and on chromogenic medium TBX agar (Oxoid) for the microbial count of *E. coli*. The plates were incubated at 30 and 37 °C, respectively, for total mesophilic and *E. coli* counts. The results were expressed as CFU (colony-forming units)/100 mL.

### DNA extraction

The DNeasy PowerSoil Kit (Qiagen) was used for the extraction of DNA from samples, as follows: 500 mL of water well sample and 50 mL of landfill leachate sample were previously filtered under vacuum on isopore polycarbonate membranes (Whatman) with a diameter of 0.45 μm within 8 h of the sample collection. The filters were aseptically cut into approximately 0.5 cm^2^ pieces and inserted into the tubes for the mechanical and chemical lysis of microbial cell walls, following the manufacturer’s protocol. Then, the extracted DNA was stored at − 20 °C prior to molecular biology analyses.

The concentration and quality of the DNA were determined by fluorometric analysis and the agarose gel electrophoresis. For the fluorometric analysis, the Qubit 2.0 Fluorometer and dsDNA HS Kit (Thermo Fisher Scientific) were used.

### PCR and target organisms

The detection and amplification of *Bacteroides*/*Prevotella* spp., *Bifidobacterium* spp., and *Enterococcus* spp. (including eight species of different human/animal origin: *E. avium*, *E. gallinarum*, *E. saccharolyticus*, *E. faecium*, *E. faecalis*, *E. hirae*, *E. casseliflavus*, and *E. durans*) were performed to identify possible fecal contamination sources. The references to the PCR methods are reported in Table 1. Furthermore, to evaluate the possible origin of *Bacteroides* spp. identified among our sample positive results and discriminate specific gene sequences for *Bacteroides* and *Bifidobacterium* strains of bovine origin (222 bp–313 bp, respectively) rather than human (119 bp–142/152 bp, respectively), the PCR products obtained in the amplification underwent enzymatic digestion with the restriction enzyme *HaeIII* (Bernhard & Field, 2000). As a positive control, a DNA sample taken from an urban wastewater treatment plant, naturally rich in *Bacteroides* spp. of human origin, was used. For *Enterococcus faecalis* and *faecium*, DNA from a collection strain of *Enterococcus faecalis*, DSMZ 2570, acted as a positive and negative control for the first and second gene amplifications, respectively.

**Table 1:** Primers set for end-point PCR of each of the target microbial groups

Primer set	Name	Sequence	Amplicon size (bp)	Reference
*Bacteroides/Prevotella* spp.	Bac32F	AACGCTAGCTACAGGCTT	676	Fiksdal et al. (1985); Bernhard and Field (2000); A. Layton et al. (2006)
	Bac708R	CAATCGGAGTTCTTCGTG		
*Bifidobacterium* spp.	Bif164F	GGGTGGTAATGCCGGATG	453	Fiksdal et al. (1985); Bernhard and Field (2000); A. Layton et al. (2006)
	Bif601R	TAAGCGATGGACTTTCACACC		
*Nitrobacter* spp.	NitroBF	TTTTTTGAGATTTGCTAG	297	Degrange and Bardin (1995); Dionisi et al. (2002); Cébron and Garnier (2005)
	NitroBR	CTAAAACTCAAAGGAATTGA		
*Nitrospira* spp.	NitrospF	CCTGCTTTCAGTTGCTACCG	151	Degrange and Bardin (1995); Dionisi et al. (2002); Cébron and Garnier (2005)
	NitrospR	GTTTGCAGCGCTTTGTACCG		
*Enterococcus avium*	AV1	GCT GCG ATT GAA AAA TAT CCG	361	B. A. Layton et al. (2010)
	AV2	AAG CCA ATG ATC GGT GTT TTT		
*Enterococcus casseliflavus*	CA1	TCC TGA ATT AGG TGA AAA AAC	269	B. A. Layton et al. (2010)
	CA2	GCT AGT TTA CCG TCT TTA ACG		
*Enterococcus durans*	DU1	CCT ACT GAT ATT AAG ACA GCG	286	B. A. Layton et al. (2010)
	DU2	TAA TCC TAA GAT AGG TGT TTG		
*Enterococcus gallinarum*	GA1	TTA CTT GCT GAT TTT GAT TCG	190	B. A. Layton et al. (2010)
	GA2	TGA ATT CTT CTT TGA AAT CAG		
*Enterococcus hirae*	HI 1	CTT TCT GAT ATG GAT GCT GTC	186	B. A. Layton et al. (2010)
	HI 2	TAA ATT CTT CCT TAA ATG TTG		
*Enterococcus saccharolyticus*	SA 1	AAA CAC CAT AAC ACT TAT GTG	350	B. A. Layton et al. (2010)
	SA 2	GTA GAA GTC ACT TCT AAT AAC		
*Enterococcus faecalis*	FL 1	ACT TAT GTG ACT AAC TTA ACC	360	B. A. Layton et al. (2010)
	FL 2	TAA TGG TGA ATC TTG GTT TGG		
*Enterococcus faecium*	FM 1	GAA AAA ACA ATA GAA GAA TTA T	214	B. A. Layton et al. (2010)
	FM 2	TGC TTT TTT GAA TTC TTC TTT A		
*Archaeal AmoA*	Amo19F	ATGGTCTGGCTWAGACG	624	Leininger et al. (2006)
	CrenamoA16r48x	GCCATCCABCKRTANGTCCA		Schauss et al. (2009)
*Bacterial AmoA*	Amo1F	GGGGTTTCTACTGGTGGT	500	Schauss et al. (2009)
	AmoA2R	CCCCTCKGSAAAGCCTTCTTC		
*Nirk*	nirK876C	ATYGGCGGVCAYGGCGA	164	Harter et al. (2014)
	nirK1040	GCCTCGATCAGRTTRTGG		
*Nosz*	nosZ2F	CGCRACGGCAASAAGGTSMSSGT	267	(Henry et al. (2006)
	nosZ2R	CAKRTGCAKSGCRTGGCAGAA		
*Nirs*	nirscd3af	AACGYSAAGGARACSGG	425	ThrobÃ¤ck et al. (2004)
	nirsR3cd	GASTTCGGRTGSGTCTTSAYGAA		

A second set of PCR analyses was conducted to evaluate the potential influence of soil nitrification or leachate contamination in the presence of nitrites and/or nitrates in the groundwater. *Nitrobacter* spp. and *Nitrospira* spp., ammonium-oxidizing archaea (AOA), and ammonium-oxidizing bacteria (AOB) were the target microbial groups. For *Nitrobacter* spp. and *Nitrospira* spp., DNA extracted from a sludge nitrification tank of a wastewater treatment plant was used as a positive control. AOA and AOB were detected from digested and purified synthetic plasmids (Geneart, Thermo Fisher Scientific, Regensburg, GmbH) containing a sequence 100% homologous with the *AmoA* gene of Archaea and Bacteria, respectively, used as controls.

The thermal amplification programs used, the reaction conditions, and the primers set for all the methods and microbial populations considered in the analysis are shown in supplementary materials (Table 2), along with relative references. In all cases, Dream Taq Master Mix (Thermo Fisher Scientific) was used, and primers were synthesized by Macrogen Europe (The Netherlands).

**Table 2:** Thermal amplification protocols and reaction conditions for end-point PCR of each of the target microbial groups

	*Bacteroides* spp./*Bifidobacterium* spp.	*Enterococcus* spp.	*Nitrobacter* spp.	*Nitrospira* spp.	*AOB*/*AOA*	*NosZ*/*NirK*
	Layton et al. (2006)	Layton et al. (2010)	Cébron and Garnier (2005)	Cébron and Garnier (2005)	Schauss et al. (2009)	(Harter et al., 2014; Henry et al., 2006)
Thermal protocol	94°Cx2’	95°Cx4’	95°Cx3’	95°Cx3’	95°Cx3’	95°Cx5’
	94°Cx30’’	95°Cx30’’	95°Cx1’	95°Cx1’	95°Cx45’’	95°Cx30’’
	35X 56°Cx45’’	30X 56°Cx1’	30X 50°Cx1’	30X 50°Cx1’	30X 60°Cx45’’	30X 60°Cx30’’
	72°Cx45’’	72°Cx1’	72°Cx1’	72°Cx1’	72°Cx45’’	72°Cx30’’
	72°Cx2’	72°Cx7’	72°Cx10’	72°Cx10’	72°Cx5’	72°Cx5’
Master mix DreamTaq DNA polymerase (Thermo Fisher) *V* = 25 µL						
Buffer 10 × (µL)	2.5	2.5	2.5	2.5	2.5	2.5
DNTPs (10 mM)	0.5	0.25	0.5	0.5	0.25	0.25
Primer F (10 µM)	1	1	1	1	1	1
Primer R (10 µM)	1	1	1	1	1	1
Taq polim. 5 U/µL	0.3	0.25	0.3	0.3	0.25	0.25
DNA	2	2	2	2	2	2
dH_2_O	17.7	18	17.7	17.7	18	18

#### PCR-ARISA

The PCR-ARISA reaction was performed according to the method of Cardinale et al. (2004) using 0.75 μL of 0.25 mM (each) ITSF (5′-GTCGTAACAAGGTAGCCGTA-3′)/ITSReub (5′-GCCAAGGCATCCACC-3′) primers, targeting bacterial ITS (Cardinale et al., 2004), in a reaction mixture containing 5 μL of 5X PCR buffer, 0.25 μL of 1.5U Taq DNA polymerase (Phusion HF DNA Polymerase—Thermo Fisher Scientific), and 0.5 μL of 0.2 mM (each) deoxynucleoside triphosphate and PCR grade water in a final volume of 25 μL and performed 35 times. Primer ITSReub was 5′-labeled with HEX fluorochrome in order to detect the ITS fragments.

The results obtained by the reaction were visualized by the 1.5% agarose gel electrophoresis run and then through a VersaDoc transilluminator (Bio-Rad, Hercules, Ca, USA).

After agarose-gel electrophoresis, the PCR ARISA products were quantified at a Qubit 2.0 fluorimeter and sent to the fragment analysis service provided by STAB-VIDA (Caparica, Portugal) to be subjected to capillary electrophoresis.

The data were analyzed using the Peak Scanner v1.0 software program (Applied Biosystem), according to the methods of Brusetti et al., 2018.

### Chemical and isotopic analyses

Chemical and isotopic compositions in groundwater and leachate followed standard procedures: (a) ion chromatography (IC) for anions (B^−^, F^−^, Br^−^, Cl^−^, SO_4_^2−^, NO_3_^−^, and NO_2_^−^) and ammonium (NH_4_^+^); (b) volumetric titration for HCO_3_^−^; (c) ICP-OES spectrometry for K^+^, Na^+^, Ca^2+^, and Mg^2+^; (d) Wavelength-Scanned Cavity Ring Down Spectroscopy technology for stable isotope values of δ^18^O and δ^2^H (the uncertainty of the measurements is ± 0.2 d ‰ for δ^18^O and ± 1 d ‰ for δ^2^H); (e) mass spectrometry IRMS with a Finnigan MAT250 for the isotopic ratio δ^13^C_DIC_ (the uncertainty of the measurements is ± 0.2 δ ‰); (f) liquid scintillation counting (LSC) for ^3^H level (the analytical precision for tritium was 0.5 TU, 1r criterion/analytical errors); (g) IRMS (Finnigan MAT 250) for δ^15^N-NO_3_^−^ and δ^18^O-NO_3_^−^ (the 1σ analytical precisions for δ^15^N-NO_3_^−^ and δ^18^O-NO_3_^−^ are ± 0.5‰ and ± 1‰, respectively). The isotopic content of δ^15^N-NO_3_^−^ and δ^18^O-NO_3_^−^ was determined also considering the main used commercial fertilizers, using the result of land use analysis (Cossu et al., 2018; Zuffianò et al., 2018).

### Statistical analyses

The cluster analysis was carried out using the number and position of the ARISA peaks of the samples as an index of the presence or absence of a given taxon and the height of the corresponding peaks as an index of the abundance of each taxon (Brown et al., 2005; Hewson & Fuhrman, 2006). From these data, a matrix was obtained by measuring the dissimilarity using the Bray–Curtis algorithm and then applying the Jaccard index. Non-metric multidimensional scaling (NMDS) analysis was realized by Past 4.07 software (Hammer et al. 2001), using the Bray–Curtis distance matrix calculated from PCR-ARISA. Environmental variables that included chemical and microbiological analyses were represented by vectors in the 2D plot generated. Diversity indexes were also calculated based on PCR-ARISA data (Brusetti et al. 2018), namely, Shannon–Wiener diversity, dominance, and evenness.

## Results and discussion

### Chemical characterization

Groundwater on-site physical and chemical parameters are summarized in Table 3. As expected, the leachate had consistently higher pH (8.2) and EC (12,347.0 mS/cm) values than groundwater. Among the groundwater samples, few differences were found: the sample of well 14 differed from others by a higher level of EC and lower DO. More generally, the EC values showed higher values in wells with a high discharging rate and/or with a higher boring depth, as is usual in a coastal aquifer, for the upcoming effect of seawater intrusion.

**Table 3:** On-site measurements of main physical–chemical features of groundwater samples. EC was estimated at 25 °C

Location (well)	Depth (m)	EC (mS/cm)	*T (°)*	pH (-)	DO (mg/L)	Eh (mV)
2	318	1.477	16.68	7.58	5.45	345
3	277	1.019	16.60	7.54	5.83	291
5	289	0.888	16.56	7.51	5.59	345
8	452	0.869	16.83	7.46	4.76	346
11	198	0.952	16.45	7.34	5.61	135
12	250	1.047	16.49	7.40	4.47	NP^(a)^
13	349	1.640	16.97	7.32	7.54	121
14	250	3.170	17.90	7.14	1.39	142
15	250	1.155	17.03	7.30	8.03	108

^(a)^*NP*, not performed

Fresh groundwater is characterized by variable chemical compositions (Moujabber et al., 2006), and its EC and salinity could be influenced by rainfall and high recharge events, seawater intrusion, evapotranspiration (for shallow aquifers), and anthropogenic effects due to land use and urbanization (Polemio & Zuffianò, 2020).

The DO values observed were consistent with those normally found in fresh groundwater, which is rich in DO, both due to the infiltration of meteoric water and the enrichment in the unsaturated area, as is common in this aquifer (Polemio, 2016).

Table 4 summarizes the results of the analyses of the main cations and anions determined for the groundwater and the leachate. Regarding inorganic nitrogen content, the nitrate concentration of groundwater fell in the range of 17.2–56.0 mg/L; the parameter exceeded the limit value of 50 mg/L as NO_3_^−^ (European Directive 91/676/EEC) only in well 3.

**Table 4:** Main ions concentration in sampled groundwater and leachate (all data are expressed in mg/L)

Location (well)	K^+^	Na^+^	Ca^2+^	Mg^2+^	B^−^	F^−^	Br^−^	Cl^−^	SO_4_^2−^	NH_4_^+^	NO_3_^−^	NO_2_^−^	HCO_3_^−^
2	5.3	116.2	99.9	58	0.03	< 0.1	0.5	179.6	48.8	< 0.1	36.8	< 0.1	520
3	2.8	48.7	121.4	28.1	0.02	< 0.1	0.2	92.2	13.6	< 0.1	56.0	< 0.1	410
5	2.2	27.7	90.9	46	0.03	< 0.1	< 0.1	55.3	11.1	< 0.1	33.7	< 0.1	440
8	2.4	27.3	86.3	48.3	0.02	< 0.1	0.4	49.2	13.2	< 0.1	20.9	< 0.1	455
11	2.1	27.3	112.8	49.2	0.02	< 0.1	< 0.1	52.8	16.9	< 0.1	40.9	< 0.1	510
12	2.7	44.8	109.7	53.8	0.02	< 0.1	< 0.1	76.8	18.0	< 0.1	33.7	< 0.1	540
13	5.2	131.8	120.3	65.8	0.05	< 0.1	1.1	182.6	33.8	< 0.1	32.9	< 0.1	675
14	16.1	438.9	137.3	107.2	0.14	< 0.1	1.6	389.7	105.0	< 0.1	17.2	< 0.1	1325
15	3.5	33.6	126.4	56.3	0.02	< 0.1	< 0.1	132.5	6.5	0.30	38.8	< 0.1	495
Leachate	1883.1	2134.6	65.6	53.6	1.43	2.1	2.4	2464.4	53.3	101.9	27.5	< 0.1	5035

NO_2_^−^ values were always below detectable levels (< 0.1 mg/L), while NH_4_^+^ was below the detection limit and sporadically detected in groundwater samples (< 0.5 mg/L of ammonia nitrogen as the ammonia limit value) and at high levels in landfill leachate. Mono and divalent ions were found to be considerably different from leachate to groundwater, being Na^+^, K^+^, SO_4_^2−^, and Cl^−^ significantly higher in leachate, while Ca^2+^ and Mg^2+^ were lower.

**Fig. 2:**
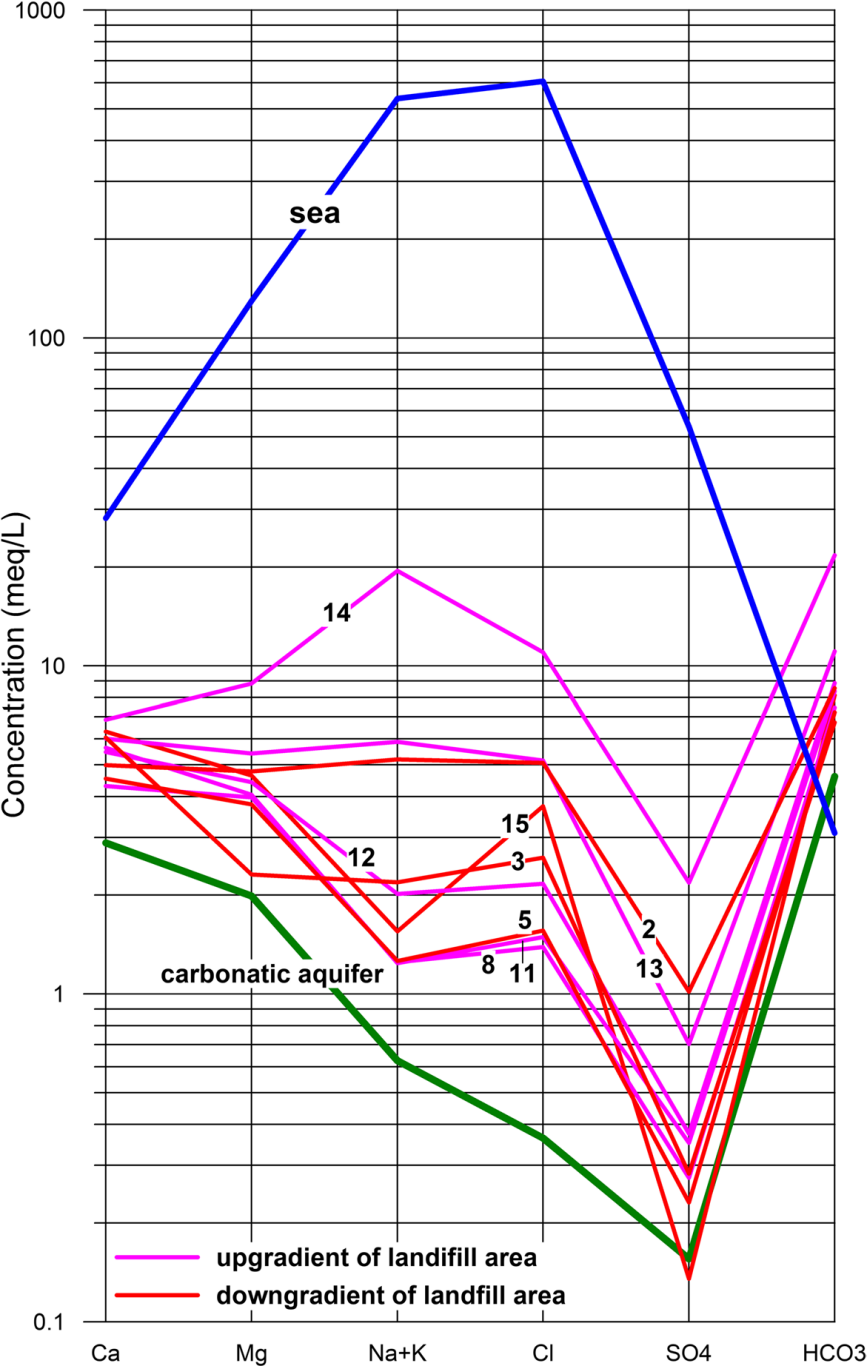
Schoeller diagram, reporting main ions composition of sampled groundwater

The distribution of the main ion concentrations in the sampled waters is shown with a Schoeller diagram (Fig. 2). The geochemical features of the samples are compared with two typical reference compositions (Polemio et al., 2009): seawater and pure fresh groundwater of the carbonate aquifer, meaning they are not affected by the seawater intrusion (sampled in the recharge aquifer area, outside and upward of the study area).

The relative abundance of major ions was mainly Ca^2+^  > Mg^2+^  > Na^+^  + K^+^ for cations and HCO_3_^−^ > Cl^−^ > SO_4_^2−^. The groundwater within carbonate aquifers is generally characterized by a predominance of calcium and bicarbonate ions due to the dissolution of calcite and dolomite. The leachate sample has completely different geochemical characteristics from those of groundwater samples (Table 4).

The results of the isotopic composition are shown in Table 5. Stable isotopic compositions, comparable for all groundwater samples and completely different with respect to leachate samples, range from − 6.53 to − 6.42‰ for δ^18^O and from − 42.89 to − 39.13‰ for δ^2^H in groundwater, while the value is − 4.29‰ for δ^18^O and 4.70‰ for δ^2^H in leachate.

**Table 5:** Isotopic composition of groundwater and leachate

Sample	δ^18^O (‰SMOW)	δ^2^H (‰SMOW)	δ^13^C_DIC_ PDB	Tritium (TU)	d-Excess (‰)	δ^15^N_NO3_ (‰air)	δ^18O^_NO3_ (‰VSMOW)
2	− 6.65	− 40.63	− 9.67	< 0.6	12.56	2.84	6.17
3	− 6.47	− 39.13	− 11.88	0.8 ± 0.4	12.66	3.37	8.52
5	− 6.55	− 40.32	− 9.86	0.6 ± 0.3	12.09	3.93	7.57
8	− 6.77	− 41.29	− 9.17	< 0.6	12.86	5.22	8.12
11	− 6.58	− 40.60	− 10.53	1.1 ± 0.4	12.04	4.37	7.36
12	− 6.61	− 40.82	− 9.73	0.8 ± 0.3	12.03	4.61	7.93
13	− 6.66	− 40.58	− 9.09	< 0.6	12.72	3.34	7.68
14	− 6.65	− 40.89	− 7.44	< 0.6	12.33	6.78	9.56
15	− 6.53	− 40.40	− 9.58	1.7 ± 0.4	11.87	10.92	8.02
Leachate	− 4.29	4.70	23.24	231.6 ± 7.3	38.98	7.62	NP^(a)^

^(a)^*NP*, not performed

**Fig. 3:**
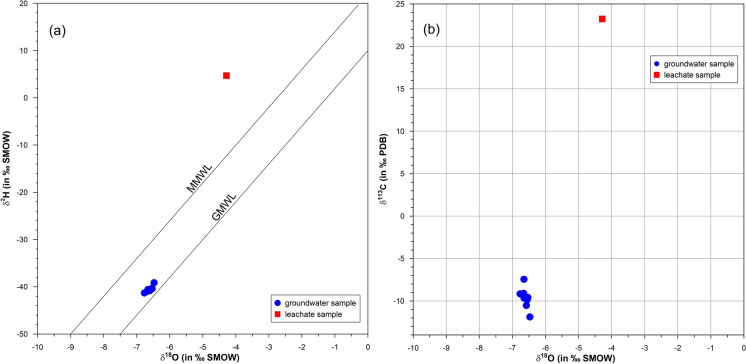
** a** Binary δ^2^H – δ^18^O diagram for water samples and leachate. **b** Binary δ^13^C – δ^18^O diagram

Figure 3a shows the δ^18^O/δ^2^H values compared with the Global Meteoric Water Line (GMWL; Craig, 1961) and the Mediterranean Meteoric Water Line (MMWL; Gat & Carmi, 1970).

The δ^18^O/δ^2^H diagram shows that the samples of groundwater are closer to the Global Meteoric Water Line than the leachate data. This indicates a rapid infiltration of meteoric water to recharge coastal aquifers in a temperate climate.

The absence of a linear distribution of the points prevents the reconstruction of a Local Meteoric Water Line (LMWL), also denoting that the sampled groundwater belongs to a geographically limited area, likely coming from the same aquifer with the same recharge area.

The isotopic variability of δ^13^C in groundwater is rather narrow (− 7.44 ÷  − 11.88‰, Table 5, Fig. 3b) and is characteristic of uncontaminated groundwater flowing in carbonate aquifers (Clark & Fritz, 1997). In the leachate sample, the concentration is 23.24‰ (Table 5). The positive δ^13^C value and deuterium enrichment in leachate are attributed to the process of methanogenesis (Grossman, 1997; Hackley et al., 1996; Wimmer et al., 2013).

Leachate differs markedly from groundwater for the tritium content, which shows a value of 231.6 TU, while for groundwater, the maximum value is 1.7 TU.

The groundwater isotopic compositions of dissolved nitrates range between + 2.84 and + 10.92‰ vs. AIR in δ^15^N-NO_3_^−^ and between + 6.17 and + 9.56‰ vs SMOW in δ^18^O-NO_3_^−^ (Table 5).

### Microbiological characterization

Microbial counts and PCR detection of the different target microorganisms are summarized in Table 6. The total mesophilic count of the groundwater showed average values of 512 (± 1.019) CFU/100 mL, with values ranging from 3 (well 13) to 3200 (well 15). *E. coli* was not isolated in any groundwater sample, except for well 14, where the count was 3 CFU/100 mL. The mesophilic bacterial count for all the groundwater samples was in line with what was reported in the literature in other studies (Keesari et al., 2015) and consistent with the range assessed in a regional study focused on the regional area that was studied in the present work (De Giglio et al., 2016). Regarding the fecal indicator *E. coli*, it should ideally not be present in the groundwater sample, as it is an indicator of recent fecal contamination. Considering that only one groundwater sample was found positive for *E. coli*, with a low count and according to the other fecal contamination microbial markers (as described below in the paragraph), it can be stated as sporadic contamination. In a previous study on a different landfill site, Grisey et al. (2010) reported a higher level of fecal coliforms and enterococci in the inspection wells than in the landfill leachate itself, due to the contamination of a septic tank of the toilet system located in the landfill plant.

**Table 6:**
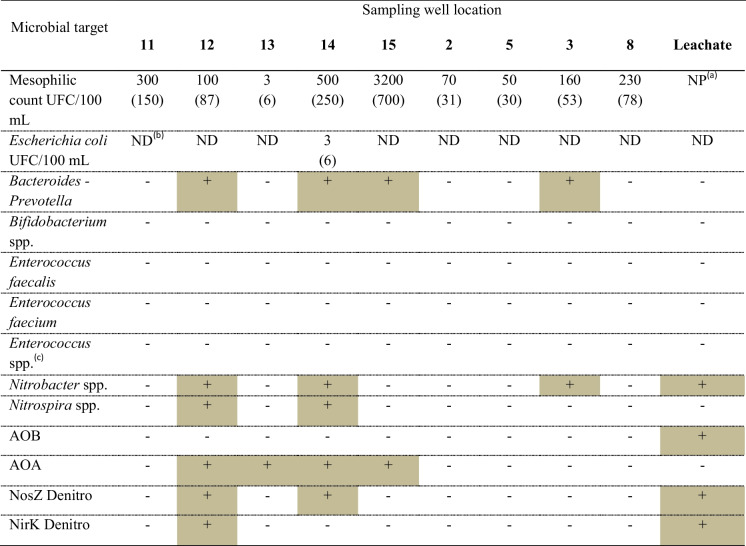
Summary of the plate counts and PCR results targeting microbial indicators. Standard deviations are reported in parenthesis

^(a)^*NP*, not performed. ^(b)^*NP*, not detected in 100 mL. ^(c)^Negative test for all six different molecular markers targeting 6 species belonging to the genus *Enterococcus*

**Fig. 4:**
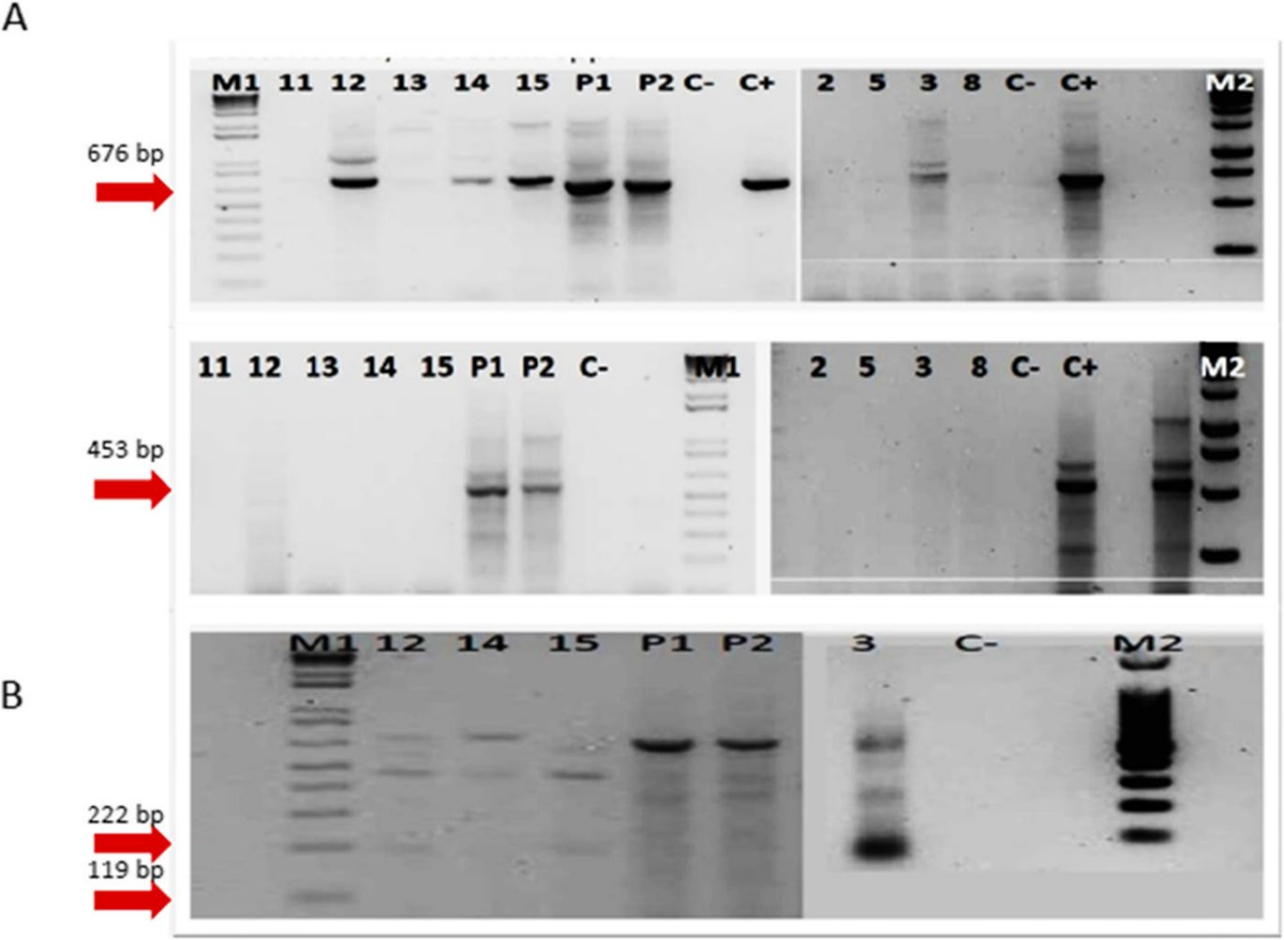
**A** Electrophoretic PCR visualization realized with primer for *Bacteroides*/*Prevotella* spp. (16SrDNA target gene, expected amplicon size 676 bp, and *Bifidobacterium* spp., expected amplicon size 453 bp). M1 = molecular weight marker 1 kb (Invitrogen), Samples of wells 11, 12, 13, 14, and 15; P1, P2 = DNA extracted from leachate coming from the landfill; C −  = no template control (white); C +  = DNA extracted from purifying sludge; M2 = molecular weight marker 1 Kb (Promega); 2–5 = samples of wells 2, 5, 3, and 8; C −  = no template control (white); C +  = positive DNA control from sewage sludge. **B** Electrophoretic visualization of enzymatic digestion with *HaeIII* on PCR products of *Bacteroides* spp. positive samples. (16SrDNA target gene, expected fragment size for human/bovine, 119/222 bp)

The PCR targeting indicators of fecal contamination of the groundwater showed the following results: *Bacteroides* spp. was detected in well samples 12, 14, and 15 since a target amplicon of 676 base pairs was generated. Similarly, the amplicon produced by the leachate sample had a molecular weight comparable to what was expected for *Bacteroides* spp. Subsequent enzymatic digestion with *HaeIII* of the above-reported amplicons generated non-specific 190 bp and 460 bp fragments for wells samples 12, 14, and 15, while no fragment was produced for leachate DNA from the starting PCR product (Fig. 4).

**Fig. 5:**
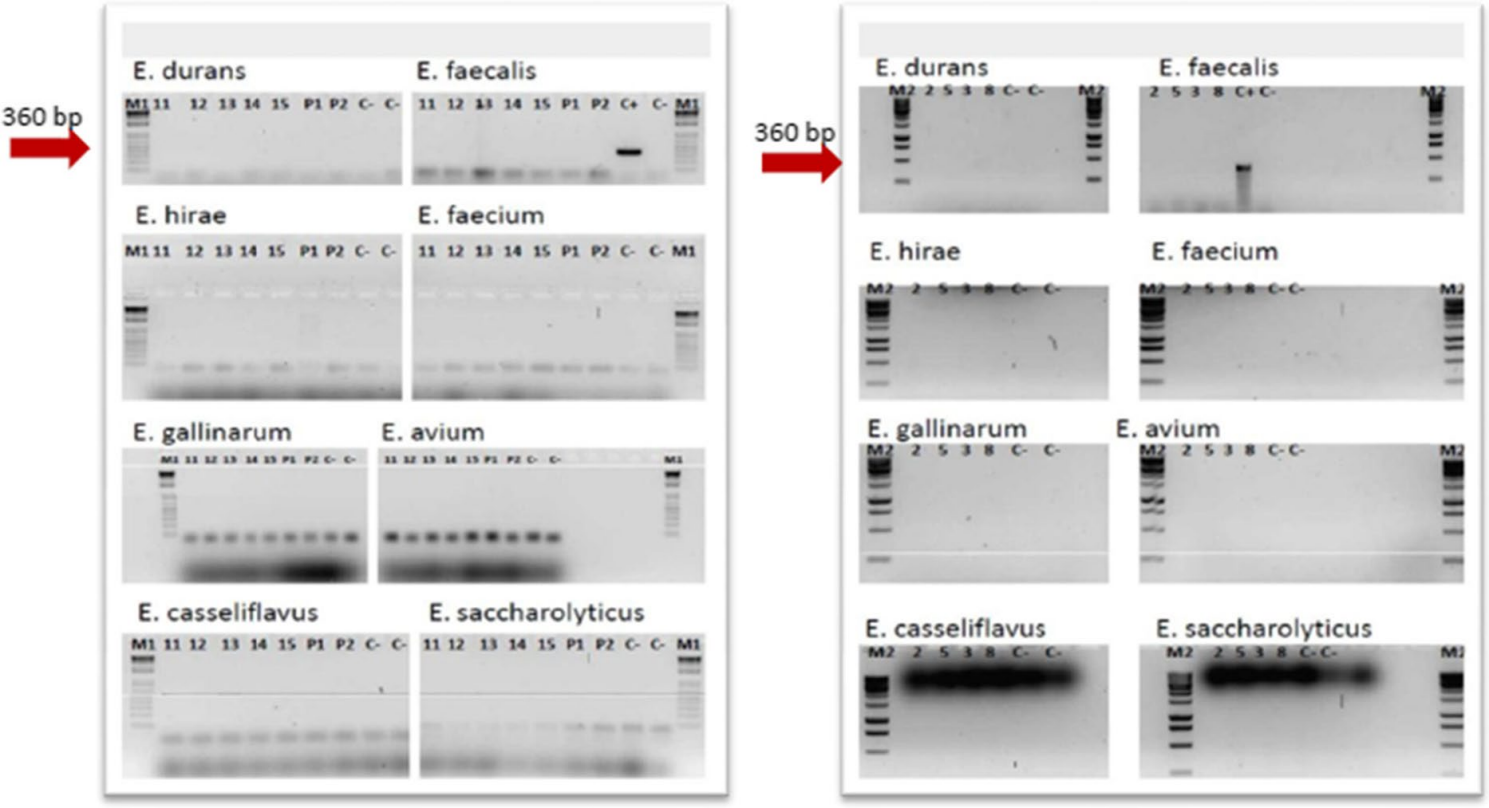
Electrophoretic visualization of PCR carried out on all samples of the 9 wells for the research of 8 different *Enterococcus* species

No samples were found positive for *Bifidobacterium* spp., since the PCR targeting eight different species of *Enterococcus* spp. related to specific animal sources was negative for all groundwater samples and for the leachate (Fig. 5).

Therefore, a general confirmation of the absence of markers of fecal contamination was observed. The only exception was the *Bacteroides*/*Prevotella*. We adopted a method able to potentially discriminate between human or zoonotic origin of *Bacteroides* (Bernhard & Field, 2000). Despite positive results for *Bacteroides* spp. obtained from leachate and from 4 wells, the restriction profile of the positive amplicons in the wells was clearly different from the leachate. In both cases, we could not attribute a potential host, even though we could exclude the human origin for both samples since no specific restriction fragment was obtained for all the samples.

According to the microbiological analyses carried out with both cultivation and molecular methods, it is possible to exclude groundwater pollution linked to fluid urban waste (e.g., wastewater treatment plants and sewage pipes) or intensive breeding and manure in the sampled site. Similarly, it is possible to exclude the hypothesis of an intake of fecal microorganisms by landfill leachate.

A second set of PCR assays (Table 6) was made to detect nitrogen cycling-related microorganisms, particularly ammonia-oxidizing, nitrite-oxidizing, and denitrifying, as indicators of the occurrence of nitrification–denitrification processes in the groundwater environment.

Interestingly, in the case of ammonia-oxidizing bacteria and archaea (AOB and AOA), leachate tested positive for the PCR targeting the *AmoA* (ammonium monooxygenase) gene of AOB, while all groundwater samples were negative. Conversely, the *AmoA* gene of the AOA was detected in wells 12, 13, 14, and 15 but was absent in the leachate. Wells 12, 13, and 14 are upward of both the two active landfills and four of the five district landfills (Fig. 1). These data confirmed the presence of different nitrifying communities in groundwater and leachate. A previous study also reported the dominance of nitrifying archaeal populations in groundwater environments with low ammonia levels (Zheng et al., 2017). The presence of different taxa between leachate (presence of AOB only) and groundwater (presence of AOA only) is noteworthy and underscores the absence of direct interaction between groundwater and leachate. While both environments may support nitrifying bacterial activity, the distinctive features observed in each suggest incompatibility for potential microbial contamination of water by leachate. As reported in a recent review (Meyer-Dombard et al., 2020), there is still little knowledge about nitrogen cycling gene activity in landfill environments. Although our results referred to a limited dataset, we found distinct nitrifier communities in the landfill leachate compared to the ones from all groundwater samples. Our results align with a previous study (Zhu et al., 2007) that underlined specific nitrifier populations in ammonia-rich landfill leachate environments.

**Fig. 6:**
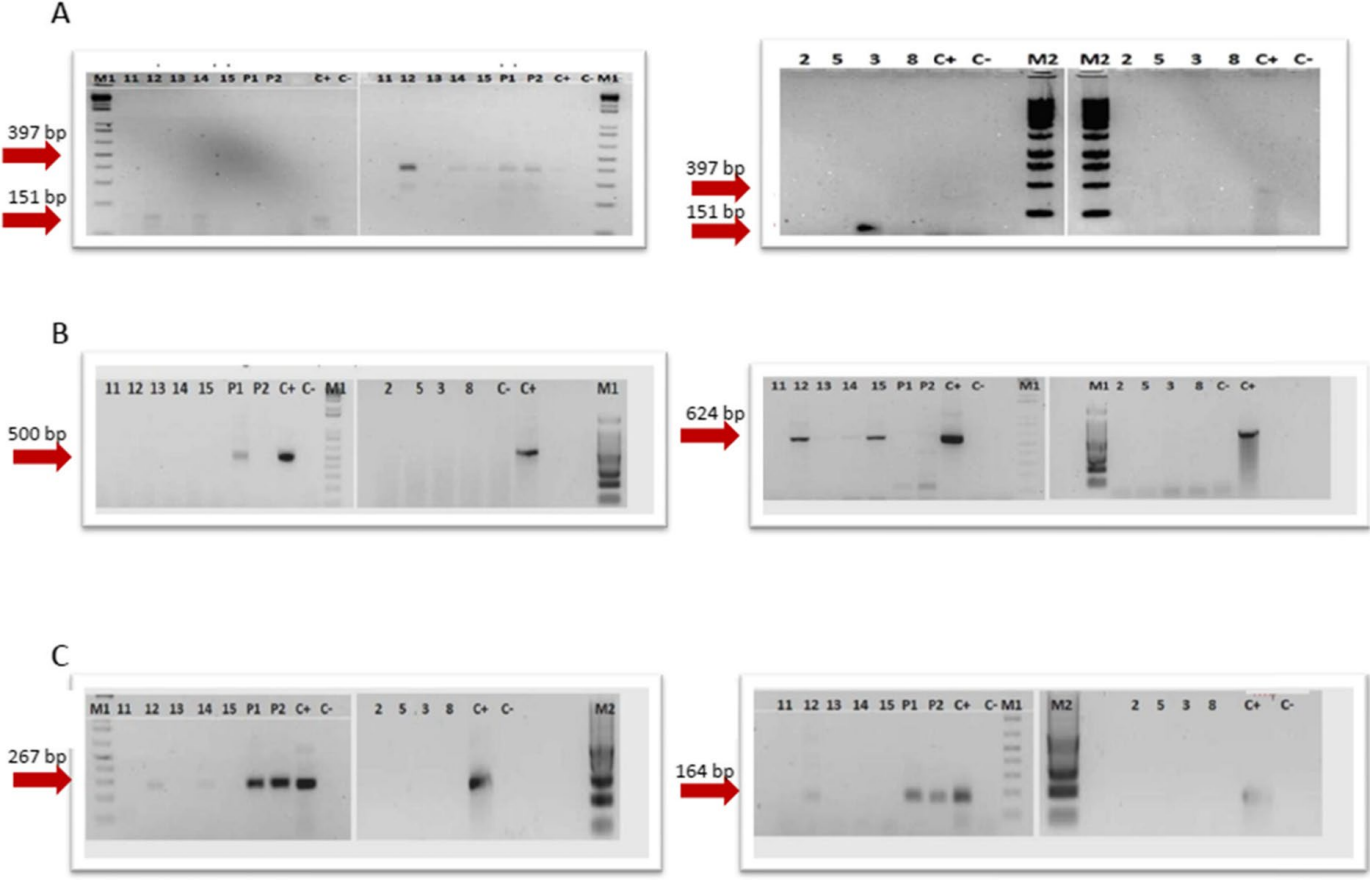
**A** Electrophoretic visualization of the PCR carried out on all the samples of the 9 wells for the research of *Nitrospira* spp. and *Nitrobacter* spp. (16SrDNA target gene, expected fragment size 397 bp for *Nitrobacter* spp. and 151 bp for *Nitrospira* spp.). **B** Electrophoretic visualization of the PCR conducted on all the samples of the 9 wells for the research of AOB and AOA. (Target gene AmoA, expected fragment size 491 bp for AOB and 624 bp for AOA). **C** Electrophoretic visualization of the PCR carried out on the samples of the examined wells for the search of denitrifying bacteria. Target NosZ genes, expected fragment dimensions 267 bp and NirK, and expected fragment dimensions 164 bp

About nitrite-oxidizing communities, samples from wells 12 and 14 were positive for both *Nitrobacter* spp. and *Nitrospira* spp., while samples from wells 15 and 3 and the leachate were positive for *Nitrobacter* spp. only (Fig. 6). It is assumed that the origin of these nitrifying microorganisms is attributable to the surrounding soil or soil fertilizers applied near the wells, considering that the chemical analysis showed a null intake of nitrites in the groundwater.

Finally, the search for denitrifying microbial populations completed the evaluation of the potential origin and fate of the inorganic nitrogen forms present in the area examined. Denitrifiers were found in the leachate, as well as in wells 12 and 14. Other groundwater samples were all negative, with none showing a positive result for the *NirS* gene (data not shown). The functional role of denitrifiers’ community both in groundwater and landfill leachate environments are complex and very different, as reported in previous studies (Cao et al., 2019; Cerminara et al., 2020; Heffernan et al., 2012; Utom et al., 2020). In our study, we can confirm that leachate hosts a denitrifying population that is compatible with a reducing environment in which nitrate can be used as an alternative electron acceptor by denitrifiers. The denitrifying population presence is negligible in the groundwater, stressing the inconsistency of the hypothetical contamination of groundwater by landfill leachate. It is important to note that denitrification is a process observed both in natural soil and instances of excessive nitrate intake after leaching, whether in water or through percolating leachate from MSW. These phenomena are not interrelated, and an adequate supply of organic carbon and a limited presence of oxygen can occur in the presence of high amounts of nitrates, regardless of their source of origin. Given the limited number of samples analyzed in the present study, we cannot make further assumptions about their ecological role.

The similarity matrix generated from the PCR-ARISA fragment of bacterial communities was used for cluster analysis, to evaluate possible influence of leachate on the bacterial communities of the aquifer.

**Fig. 7:**
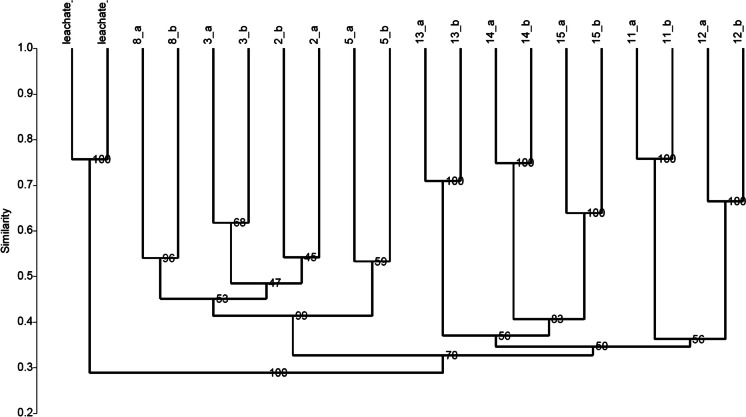
Cluster analysis based on ARISA patterns obtained from bacterial community of the groundwater samples (well number replicates a–b) and the leachate. Bray–Curtis similarity index is reported on the axis. The three clusters are numbered with roman numbers. Numbers on the nodes are the results of bootstrap analysis (5.000 repetitions)

The ARISA profiles of bacterial communities (Fig. 7) revealed that three clusters are clearly distinguishable (reported with brackets and Roman numbers) and evidence a major difference in bacterial communities in different sites of the sample area.

Particularly, the wells close to the main group of landfills, named 11–12-13–14 (internal to the landfill district) and 15 (downward of the district), clustered together (cluster I). Cluster II grouped the microbial community of groundwater sampled from wells 2, 5, 3, and 8, located far from the landfill district, both upward and downward. Interestingly, the leachate bacterial community was found to be highly dissimilar to all the groundwater samples and grouped separately in a specific cluster (cluster III). The microbial communities of the wells are strictly influenced only by their location in space and human activity (anthropized and non-anthropized), without a relation to the distance or the upward or downward location with respect to the landfill district.

**Fig. 8:**
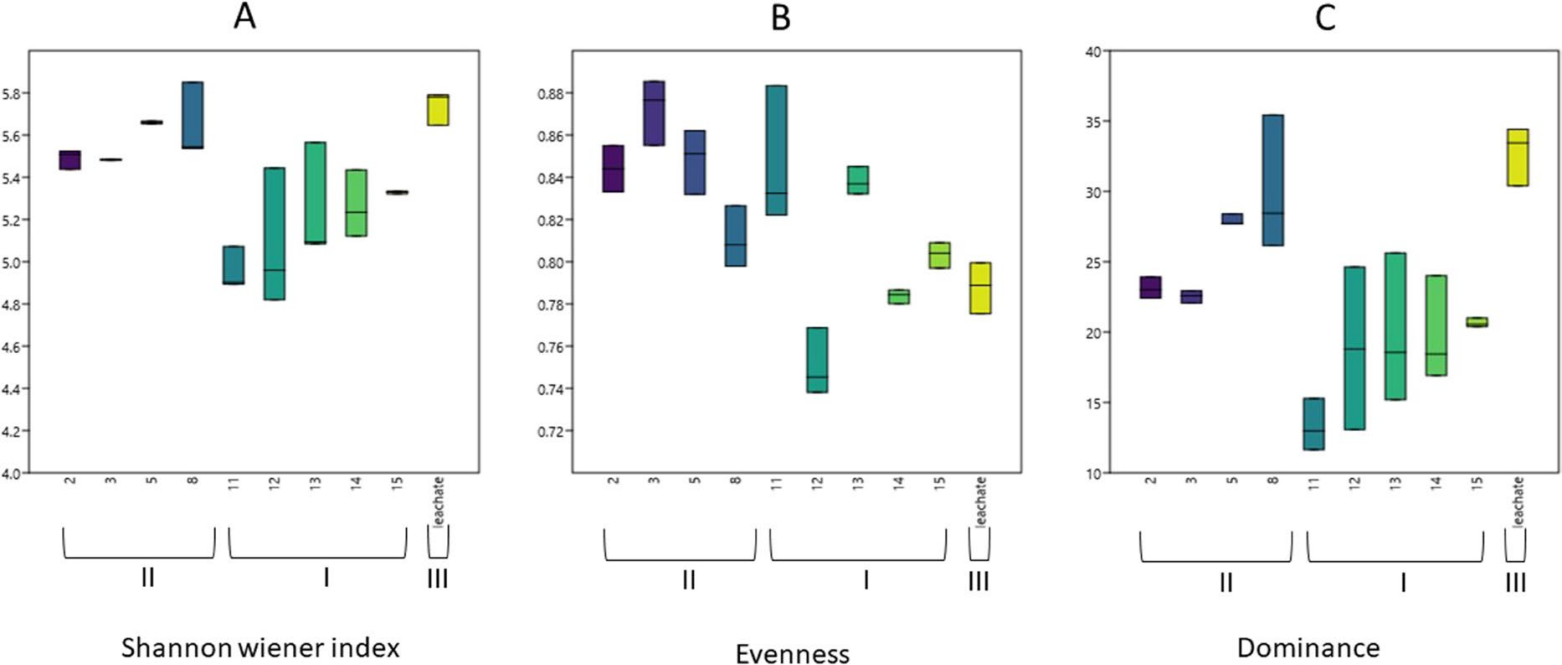
Box plot of the diversity indexes of leachate and groundwater bacterial communities, based on the ARISA analysis. Central bar represents the median, and rectangles represent first and third quartiles. **A** Shannon–Wiener, **B** evenness, and **C** dominance. Samples are grouped and numbered with roman numbers according to the clustering reported in Fig. 7

The alpha diversity analysis was also reported (Fig. 8), to evaluate the richness of the groundwater bacterial communities and the abundance variation within sampled sites.

The Shannon–Wiener diversity index (Fig. 8A) depicted a general trend of higher bacterial diversity in the wells sampled far away from the landfill district (group II), where agricultural use of the land is the exclusive activity. Wells sampled in the landfill district showed a lower diversity (group I). Despite its proximity to landfill zone inspection wells, the landfill leachate showed a higher diversity than the closest wells. The differentiation among abundance trends in the different samples was mostly explained by the evenness of the bacterial communities, as reported in Fig. 8B, C, where Shannon’s evenness and dominance indexes are reported. While the dominance was relatively low in all samples, the evenness of samples far from the landfill (group II) was considerably higher than both leachate and wells in the proximity of the landfill site, which was also highly variable. A possible explanation is that the anthropic influence of the landfill environment (concrete pavement, buildings, truck handling, etc.) may affect the underground water environment, shaping a different bacterial assembly than in the surrounding environment, where agriculture is the major activity. Leachate bacterial communities were different both in terms of alpha and beta-diversity from the groundwater sampled both close and far from the landfill site, confirming that no direct influence of potential leachate infiltration could be drawn.

### Correlation of microbial community with environmental variables

**Fig. 9:**
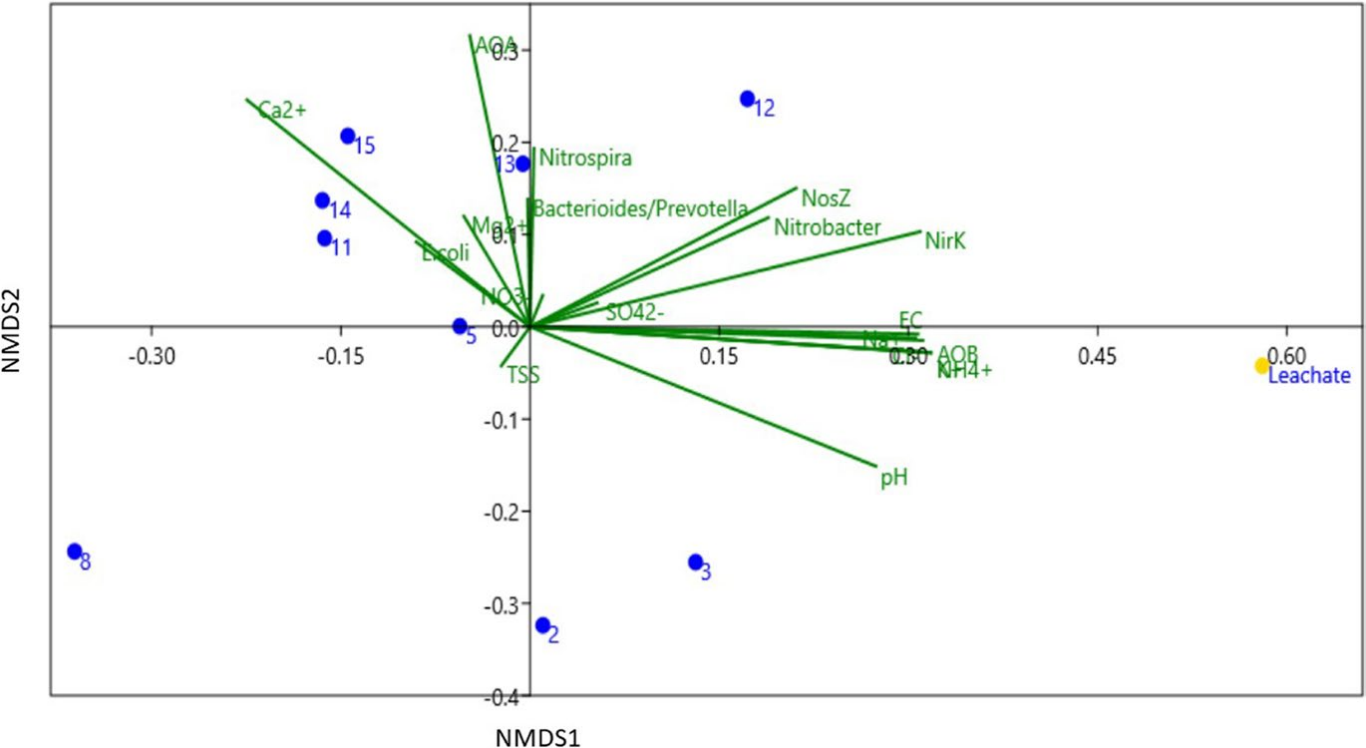
Non-metric multidimensional scaling (NMDS) ordination of the bacterial community structure, showing the relationships with environmental variables. Groundwater samples: blue dots, leachate: yellow dot

Finally, to evaluate if and how microbial groundwater communities were differentially shaped by environmental variables, a non-metric multidimensional scaling method (NMDS) analysis was also conducted (Fig. 9).

The NMDS confirms a high distance among a bacterial community assembly close to and far from the landfill site. Leachate sample coherently with previous results, clustered separately by all groundwater sample points. In particular, the figure outlined how physical parameters like EC and pH and chemical parameters (mostly EC, NH_4_^+^, B^−^, and Cl^−^) were the main drivers of leachate bacterial community differentiation in space. On the opposite, the differentiation of groundwater clusters was mainly driven by divalent cations (Mg^+^ and Ca^+^) and seawater intrusion mixing parameters (EC and Cl^−^) but it was also related to the differential presence of NO_3_^−^ and the N cycling microbial key groups (AOA, *Nitrobacter* spp., and *Nitrospira* spp.).

**Fig. 10:**
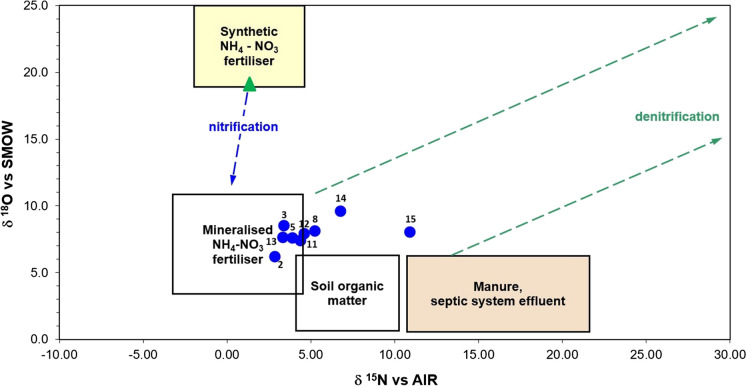
Stable isotope composition of dissolved nitrates in groundwater (blue dots) and fertilizer (green triangle). The rectangles and the oriented lines highlight the main types of nitrate sources and the effect of the processes respectively (modified after Clark & Fritz, 1997)

Figure 10 shows the values of δ^18^O and δ^15^N of the groundwater nitrate, including the “Nitrophoska Special” fertilizer, widely used in the areas around the site under study for agricultural purposes, in order to identify the different sources of nitrate. The graph shows that the isotopic delta of most groundwater samples falls to the mineralized NH_4_-NO_3_ fertilizer area. That indication confirms the hypothesis of active nitrification in the soil and its possible impact on the nitrate content of the groundwater derived from agriculture and microbial activity rather than any other possible source (Nestler et al., 2011).

The groundwater samples 14 and 15 show a higher δ^15^N-NO_3_^−^ isotopic signature as an effect of partial denitrification. Those results are coherent with previous studies on CLD that highlighted the effect of the denitrification process in the groundwater sampled (Cossu et al., 2018).

## Conclusions

Our study is aimed at proposing a multidisciplinary approach for environmental monitoring of a potentially polluted aquifer. The approach integrates chemical and isotopic analyses with both culture based and molecular microbiology methods. It offered explanations of all the measured variability in the monitored site that led to robust conclusions about absence of leachate pollution events in the surveyed period.

The proposed approach confirmed that leachate had no measurable influence on groundwater microbial community, showing other anthropic activities and seawater intrusion explain well the measured variability of the microbial populations. On the other hand, the chemical and isotopic results confirmed the absence of leachate effects on the groundwater samples, showing the decisive role of fertilizers as potential nitrate sources, as confirmed by N-cycling bacterial population features evidenced by molecular methods.

According to the results, this multi-integrated method clearly shows the absence of leachate effects on groundwater in the Conversano landfill district despite the inherent difficulty of operating in a karstic aquifer affected by seawater intrusion and with very deep groundwater. The integrated methods also give explanation of all observed variabilities in both chemical and microbiological parameters.

Compared to previous studies, the proposed approach shows advantages considering site-specific factors concerning natural ecology and potential anthropic disturbances related to landfills, agriculture, and other potential pollution sources. The next aim will be extended to a broader sampling period and different sites, providing a solid scientific basis for the effective waste management control and ecological restoration of landfills, in complex sites constituted by more landfills and including different pollution sources.
